# The Establishment of a Highly Efficient In Vitro Regeneration System for *Viburnum opulus* L. ‘Roseum’

**DOI:** 10.3390/plants14030374

**Published:** 2025-01-26

**Authors:** Yajing Ning, Hao Dong, Xinxin Zhang, Yanhua Li, Chengpeng Cui, Shujuan Li

**Affiliations:** 1 State Key Laboratory of Tree Genetics and Breeding, Northeast Forestry University, Harbin 150040, China; 2 Zhejiang Physical and Chemical Technology Co., Ltd., Hangzhou 310000, China

**Keywords:** axillary shoot proliferation, micropropagation, plant tissue culture, ornamental plants

## Abstract

*Viburnum opulus* L. ‘Roseum’ is a highly valuable ornamental plant for landscaping, but it has a long propagation cycle and low propagation coefficient. In this study, stem segments with axillary buds from *Viburnum opulus* L. ‘Roseum’ were used as explants. We systematically analyzed the use of sodium hypochlorite for the sterilization of explants, as well as the effects of different plant growth regulator combinations and concentrations on shoot bud induction, shoot proliferation, the rooting of tissue-cultured shoots, and the transplanting of the tissue-cultured shoots. A complete rapid propagation technology system for *Viburnum opulus* L. ‘Roseum’ was established. The results showed that a disinfection method using 75% ethanol for 30 s and soaking in 5% sodium hypochlorite for 5 min was the most suitable for disinfecting the stem segments of *Viburnum opulus* L. ‘Roseum’, which showed low contamination and a 73.33% survival rate. The ideal medium for primary bud induction was WPM (Woody Plant Basal Medium) + 2.0 mg·L^−1^ 6-benzylaminopurine (6-BA) + 0.15 mg·L^−1^ indole-3-butyric acid solution (IBA) + 25 g·L^−1^ sucrose. The optimal medium for shoot proliferation was WPM + 1.0 mg·L^−1^ 6-BA + 0.15 mg·L^−1^ IBA + 25 g·L^−1^ sucrose, achieving an induction rate of 7.17. For the rooting of tissue-cultured shoots, the most suitable formulation was 1/2 WPM + 0.3 mg·L^−1^ naphthaleneacetic acid (NAA) + 0.3 mg·L^−1^ activated charcoal (AC) + 25 g·L^−1^ sucrose, which induced robust and developed root systems. This study provides a technical basis for the establishment of a fast propagation system for the industrial production of *Viburnum opulus* L. ‘Roseum’.

## 1. Introduction

The *Viburnum* plant holds a very important position in ornamental plant resources due to its strong adaptability and high ornamental value, earning the title of a ‘universal greening tree’ [1]. *Viburnum opulus* L. ‘Roseum’ is a deciduous shrub of *Viburnum* [2]. It is cultivated in China in Jiangsu, Zhejiang, Jiangxi, and Hebei Provinces. The young branches of the year are ribbed and hairless on the surface. The winter buds are oval-shaped and have one pair of fused outer scales. The leaves are round-ovate, broad-ovate, or reverse-ovate, measuring 6–12 cm in length, with leaf lobes, where the middle lobes are long, palmately three-veined, and the base is round, flat, or shallowly heart-shaped, with a hairless surface. The petioles are relatively thick, measuring 1–2 cm in length, and have 2–4 or more disk-shaped glands. The compound umbel inflorescences are 5–10 cm in diameter, featuring large sterile flowers, with thick total peduncles measuring 2–5 cm in length and a hairless surface, and the flower stalks are extremely short. The flower corolla is white and radially symmetrical. The sterile flowers are also white, measuring 1.3–2.5 cm in diameter, and have long stalks, with broad and reverse-ovate lobes. The leaves of *Viburnum opulus* L. ‘Roseum’ fall in winter and sprout in early spring, remaining green until early autumn. During the transition from autumn to winter, the leaf color changes to red, making it a highly ornamental landscape plant. As an ecological landscaping tree species, *Viburnum opulus* L. ‘Roseum’ can be planted singly, in clusters, or mixed with other plants. It is suitable for use as a garden tree, street tree, or landscape tree and is also appropriate for large-scale planting as an ornamental tree in botanical gardens and natural scenic areas [2]. *Viburnum opulus* L. ‘Roseum’ seedlings are difficult to source, and there are many sterile flowers, and cutting propagation suffers from low rooting rates. Therefore, it is of great significance to study the efficient propagation of *Viburnum opulus* L. ‘Roseum’ to provide technical support for urban greening.

*Viburnum opulus* L. ‘Roseum’ is an important ornamental plant resource with strong adaptability, making it popular in landscape design. Research by Du Xiao [3] and Bi Bo [4] showed that *Viburnum tinus* and *Viburnum punctatum* have strong heavy metal accumulation capabilities. In addition to its high esthetic value, *Viburnum opulus* L. ‘Roseum’ also contributes to environmental beautification and has ecological benefits. Currently, the main propagation methods for *Viburnum opulus* L. ‘Roseum’ are cuttings and stem segments, which have a low propagation capacity. It is challenging to obtain a large number of healthy and disease-free plantlets in a short period due to the long cycle and low propagation coefficient of these methods, resulting in current production not meeting market demand. In recent years, there have been related reports on the propagation technology of *Viburnum opulus*. L. Chen Bihua [5] conducted experiments on cuttings using *Viburnum macrocephalum* Fort. as the material. The results showed that treatment with 500 ppm IBA for 5 min resulted in a rooting rate of 66.5% to 66.9% after 60 days. Bi Xianyu [6] tested cuttings of current year branches of *Viburnum Sargenti* Koehne, and the results surfaced that their survival rates ranged from 53.28% to 61.53%. Plant tissue culture can meet the demands of large-scale plant propagation and serves as an important tool for studying plant resistance to stress [7]. Virginia Hildebrandt [8] used *Viburnum opulus* ‘Nanum’ as a material and indicated that each bud could produce approximately seven explants after 6–8 months. This indicates that tissue culture is an important method for the rapid propagation of plants, allowing for both efficient reproduction and the preservation of excellent traits in clonal lines.

*Viburnum opulus* L. ‘Roseum’ has significant landscape ecological value and high market demand. Its propagation coefficient is low and its cycle is relatively long when propagated via cuttings, whereas tissue culture can yield numerous tissue-cultured plantlets in a short time. Currently, reports on the tissue culture techniques for *Viburnum opulus* ‘Roseum’ indicate a low proliferation rate. Suaad A. Yaseen [9] conducted research using *Viburnum opulus* ‘Roseum’ as the material, finding a proliferation rate of 3.31. Therefore, we aimed to establish a more efficient rapid propagation tissue culture system for robust *Viburnum opulus* ‘Roseum’ to provide an effective pathway for large-scale tissue-cultured plantlet production while better meeting the needs of production and the market.

## 2. Results

### 2.1. Surface Sterilization of Explants

The disinfection results of *Viburnum opulus* L. ‘Roseum’ stem segments treated with different concentrations of sodium hypochlorite and varying disinfection times are shown in Table 1. When a 2% sodium hypochlorite solution was used for disinfection for 5 and 10 min, the stem segments did not die but the contamination rates reached as high as 80.00% to 62.22%, with the highest survival rate being only 37.78%. In contrast, when a 5% sodium hypochlorite solution was used for disinfection for 5 min, the contamination rate decreased by 60% compared with the 2% sodium hypochlorite solution. The highest survival rate at this time was 73.33%. When the same concentration of disinfectant was used for 10 min, a mortality rate of 10.00% was observed, and the survival rate began to decline. After treatment with a 10% sodium hypochlorite solution for 5 min, the contamination rate was only 13.33%, with a mortality rate of over 50%. When the 10% sodium hypochlorite solution was applied for 10 min, the contamination rate dropped to 0.00%, but the mortality rate surged to 75.56%.

### 2.2. Primary Bud Induction Culture

**Figure 1:**
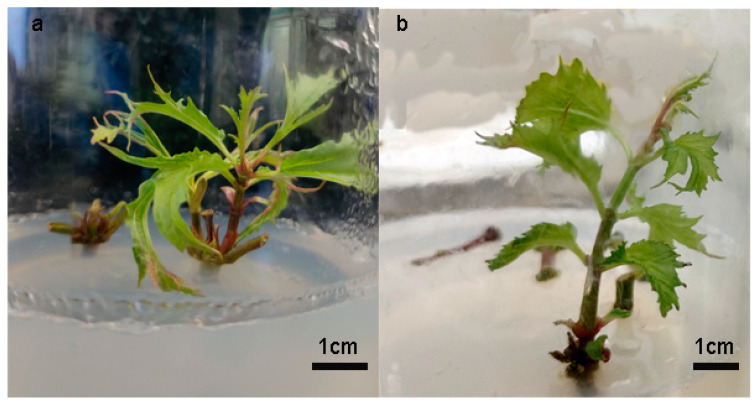
Growth status of axillary buds of the 2 initial induction schemes. (**a**) Scheme A; (**b**) Scheme B.

The stem segments that had been disinfected via explant treatment were placed in a culture medium for the initial induction of axillary buds. The germination time of the axillary buds and the state of the induced axillary buds vary among different combinations of plant growth regulators, as shown in Figure 1 and Table 2. In the initial induction Scheme A, axillary buds began to grow after 8.67 days of inoculation, whereas in Scheme B, they only started to grow after 10.33 days. After 45 days, the leaf growth rate of the axillary buds in Scheme A was fast, but the leaves were elongated, slightly curled, and had deeper leaf lobes, with the apical bud not prominent and consisting of several deformed buds. In Scheme B, the growth of the axillary buds from the stem segments was slower, the leaves were flat, and the leaf color was a vibrant green, which indicated normal axillary buds.

### 2.3. Shoot Proliferation

**Figure 2:**
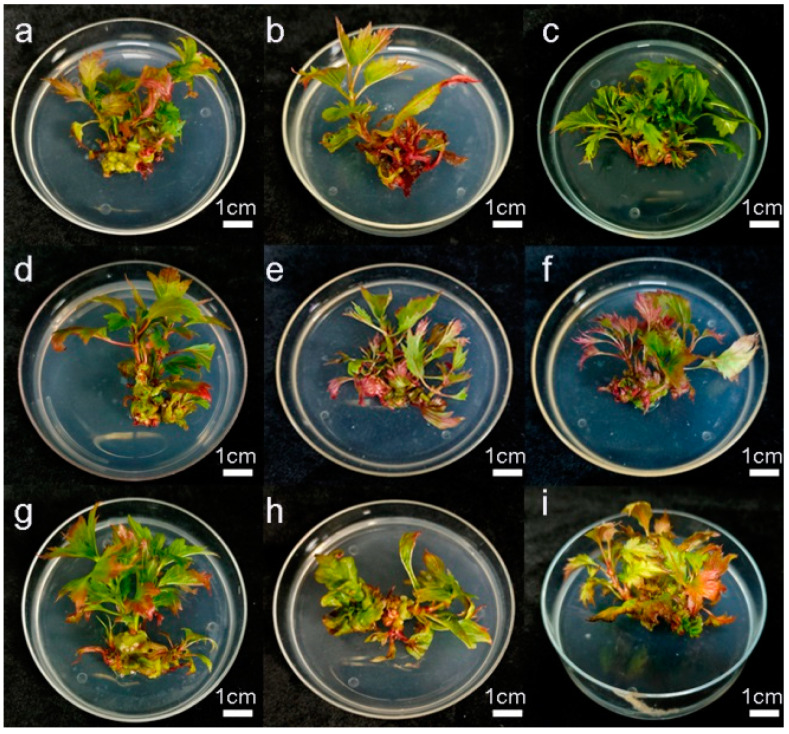
Effect of different concentrations of 6-BA, IBA, and sucrose on axillary bud differentiation. (**a**–**i**) are groups 1–9, respectively.

Table 3 and Figure 2 show that among the nine propagation schemes designed using orthogonal experiments, Scheme 3 had the highest number of axillary shoot buds (7.17), with normal growth, flat leaves, and typical leaf shapes. In contrast, Schemes 2, 6, and 8 had fewer numbers of axillary bud proliferations. In Scheme 2, the differentiated buds were generally reddish and mostly deformed. Scheme 6 produced more deformed buds, characterized by elongated leaves, deeper leaf fissures, and longer petioles. In Scheme 8, the number of axillary buds was low, with curled leaves and short internodes. Schemes 1, 4, 5, 7, and 9 produced 4.17 to 6.33 proliferated buds, but many of these exhibited reddish leaves. In Scheme 4, there was the growth of callus tissue on the stem. In Scheme 5, the proliferated buds had elongated leaves with slightly increased internodes. In Scheme 9, the buds showed poor growth, with yellowing leaves and some basal leaves dying.

### 2.4. Induction of Adventitious Roots

**Figure 3:**
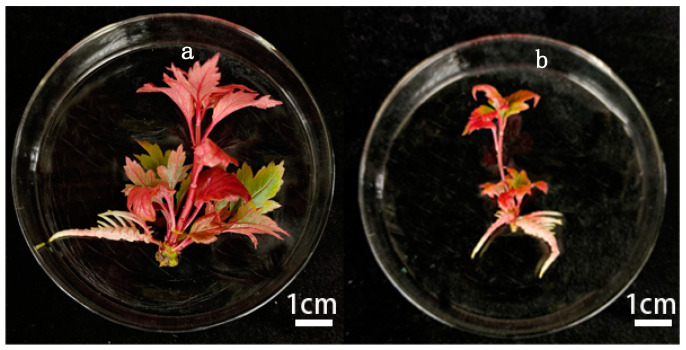
Effect of not adding AC to the medium on rooting. Both (**a**,**b**) show the state of the root system of the tissue-culture plantlets when no AC was added.

During the experiment, the tissue-cultured plantlets without added activated charcoal (AC) had less rooting, the main root was thicker, there was a whitish epidermis of the main root, and slow growth, as shown in Figure 3. However, adding an appropriate amount of AC improved the growth environment of the tissue-cultured plantlets in the medium, leading to healthier root growth. Therefore, the rooting culture phase for *Viburnum opulus* L. ‘Roseum’ tissue-cultured plantlets must include an appropriate amount of AC in the medium.

**Figure 4:**
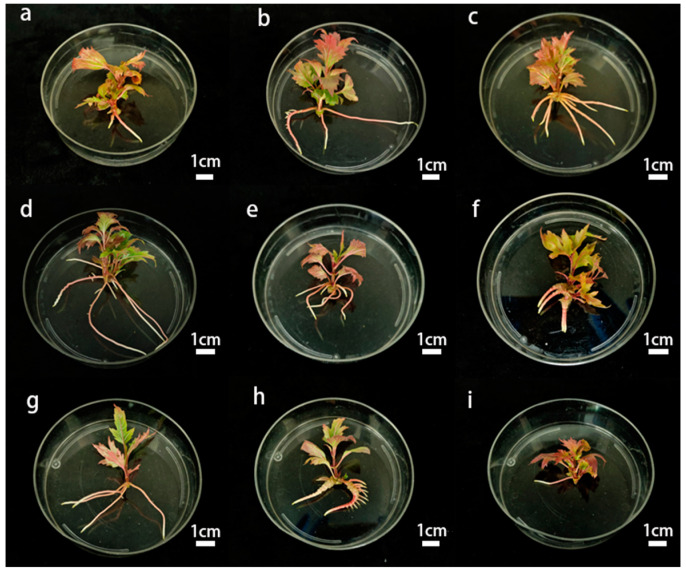
Effect of different concentrations of sucrose, NAA, and AC on rooting. (**a**–**i**) are groups 1–9, respectively.

As shown in Table 4 and Figure 4, in the nine different rooting treatments, there was still swelling in the epidermal layer of the primary roots with an AC concentration of 0.1 g·L^−1^. When the rooting regimen was 1/2 WPM + 0.3 mg·L^−1^ NAA +0.3 mg·L^−1^ AC + 25 g·L^−1^ sucrose, the tissue-cultured plantlets had a high rooting rate, relatively fast growth rate, and vigorous root system. And the root system was robust, the plants grew normally, and the leaves were flat without curling. However, as the concentration of NAA gradually increased, the rooting rate of *Viburnum opulus* L. ‘Roseum’ was inhibited, leading to slower root growth. The rooting rate of Group 3 was as high as 88.89%, followed by those of Groups 4 and 5, with a difference of only 11.11%. However, the adventitious roots of the tissue-cultured plantlets in Group 3 were robust and had a higher mean number of primary roots of 4.38, while the mean number of primary roots in Group 4 was 4.29.

### 2.5. Transplantation

**Figure 5:**
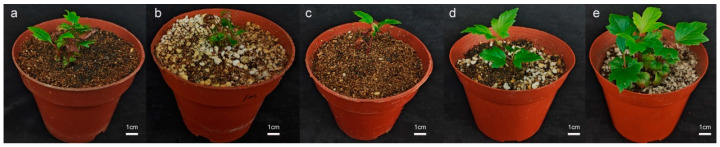
Effects of different substrates on the growth of *Viburnum opulus* L. ‘Roseum’ tissue-cultured plantlets. (**a**) Peat soil–vermiculite = 1:1; (**b**) peat soil–vermiculite–perlite = 1:1:1; (**c**) peat soil–vermiculite = 1:2; (**d**) peat soil–vermiculite–perlite = 1:2:1; (**e**) after 45 days incubation in a mixed substrate of peat soil–vermiculite–perlite at 1:2:1.

The rooting culture of *Viburnum opulus* L. ‘Roseum’ tissue-cultured plantlets was carried out based on the rooting protocol of Group 3. The tissue-cultured plantlets of *Viburnum opulus* L. ‘Roseum’ adapted to the transplanting environment after indoor acclimatization, resulting in the successful survival of all plantlets post-transplant. As shown in Figure 5, different substrates had varying effects on the transplantation of *Viburnum opulus* L. ‘Roseum’ tissue-cultured plantlets. When the tissue-cultured plantlets were cultivated in a substrate mix of peat soil and vermiculite in a ratio of 1:1 for 10 days, the surviving plantlets exhibited curled leaves that were reddish-brown, with stunted development and overall smaller stature. After 10 days of culture in a substrate mixed in a ratio of 1:1:1 of peat soil, perlite, and vermiculite, some leaves of the surviving small plants were curled, and growth was somewhat slow. When the tissue-cultured plantlets were planted in a substrate mix of peat soil and vermiculite at a ratio of 1:2 for 10 days, some leaves showed reddish-brown coloration, and the growth was somewhat slow. In the substrate mix of peat soil, vermiculite, and perlite at a ratio of 1:2:1, the tissue-cultured plantlets grew vigorously, demonstrating healthy growth and normal leaf extension. After 40 days of growth, the plants were robust, with leaves displaying a shiny appearance, and the young buds at the base also began to grow. Therefore, we preliminarily conclude that the optimal substrate for the growth of *Viburnum opulus* L. ‘Roseum’ has a ratio of 1:2:1 of peat soil, vermiculite, and perlite. In addition, the survival rate of the tissue-cultured plantlets in different substrate ratios, which only showed differences in terms of growth status, was maintained at 100%.

## 3. Discussion

Our current understanding indicates that the propagation efficiency of *Viburnum opulus* L. ‘Roseum’ is relatively low. Therefore, in this study, we established a system for the efficient in vitro culture of *Viburnum opulus* L. ‘Roseum’, comprising five stages: explant disinfection, axillary bud initial induction, multiple bud induction, rooting of shoots, and substrate selection. In sterilants, mercuric chloride and sodium hypochlorite are widely used [10]. However, mercuric chloride solution tends to leave residues on the surface of explants, causing significant damage to the explants. If not rinsed thoroughly with sterile water, it can lead to the death of the explants [11]. Therefore, in this experiment, sodium hypochlorite was selected for surface disinfection of the explants. In the experiment, after cutting the explants, they were first rinsed to remove dust and debris for 30 min under running water. Subsequently, disinfecting with 5% sodium hypochlorite for 5 min proved to be the most suitable approach for disinfection, as it not only increased the survival rate of the explants but also reduced contamination rates.

The induction rate of adventitious buds and the propagation coefficient are important indicators during the initial induction and proliferation phases and are influenced by various factors, including the culture medium and plant growth regulator [12]. Jian Defeng [13] found that the combination of KT (kinetin) and IBA plant growth regulator significantly improved propagation effectiveness in *Viburnum sargentii* Koehne. In this experiment, in the initial induction stage of *Viburnum opulus* L. ‘Roseum’, it was discovered that the medium WPM (Woody Plant Basal Medium) + 0.15 mg·L⁻^1^ indole-3-butyric acid solution (IBA) + 2.0 mg·L⁻^1^ 6-benzylaminopurine (6-BA) + 25 g·L⁻^1^ sucrose was most suitable for initial induction. When the concentration of 6-BA was 2.0 mg·L⁻^1^, the induced axillary bud leaves were deformed, and apical dominance was not evident. Wang Hongbao [14] found that a medium of MS + 0.5 mg·L⁻^1^ 6-BA + 0.2 mg·L⁻^1^ NAA was effective for *Viburnum sargentii*, with the propagation coefficient reaching 7. Wu Ju [15] discovered that the combination of 6-BA and IBA plant growth regulator was better suited for the propagation of adventitious buds in *Viburnum sargentii*, when the proliferation factor increased by up to six times. Zhen Xuehua [16] conducted tissue culture studies using young stems of *Viburnum opulus* L. as explants, based on MS (MA Base Salts) medium, achieving a multiplication rate of 2.95. In this study, it was found that the best propagation scheme used a medium of 0.15 mg·L⁻^1^ IBA + 1.0 mg·L⁻^1^ 6-BA + 25 g·L⁻^1^ sucrose, with a differentiation coefficient of 7.17 for adventitious buds. When the concentration of 6-BA increased, most of the adventitious buds became deformed, with yellowing leaves or overall reddening of the plant. The reasons for the color change in plant leaves are numerous. *Viburnum opulus* L. ‘Roseum’, as a deciduous tree species that exhibits autumn coloration, is very sensitive to changes in light and temperature. We speculate that during its cultivation, subtle variations in the external environment, as well as the effects of excessively high or low levels of plant growth regulators and carbon sources in the culture medium, lead to the formation of colored buds and malformed buds.

The rooting and acclimatization of tissue-cultured plantlets are key steps for the large-scale and commercial application of tissue culture rapid propagation technology [17]. *Ormosia henryi* Prain [18] and *Vaccinium ashei* ‘Tifblue’ [19] both utilize 1/2 WPM medium for rooting induction. Du Shurui [20] and others used IAA for the culture rooting of *Viburnum* to achieve a 93.5% rooting rate. Wu Ju [15] utilized IBA to induce rooting in *Viburnum sargentii* tissue-cultured shoots, achieving a rooting rate of 90%. In this experiment using axillary buds to induce rooting, it was found that NAA had a better induction effect, resulting in a more robust and developed root system, which is consistent with the research by Cai Neng and others [21]. The mechanism by which activated carbon promotes root formation is through its role as a physical adsorbent. Activated carbon can enhance the aeration and water-holding capacity of the culture medium, while also absorbing toxic substances secreted by the cultured material, thereby reducing the accumulation of these harmful substances. This leads to reduced oxidative stress in the root system and promotes plant root growth. Luo Mengwei [22] found that activated carbon can significantly improve the rooting rate of tissue-cultured plantlets of *Platostoma palustre*, achieving a rooting rate of 100%. Bi Xianyu [23] and others achieved a rooting rate of 90.91% by adding activated charcoal. In the present study, it was found that the non-addition of an appropriate amount of AC in the rooting medium affected the robustness of the root system of the tissue-cultured shoots to some extent. This is consistent with the findings of Bi Xianyu [6]. Roots that differentiated without AC usually had an enlarged outer epidermis with a whitish color, which was easily damaged by rinsing under running water and could easily cause root composting death of tissue-cultured plantlets during planting. Activated charcoal can provide a suitable dark environment for root development, and the addition of the appropriate amount of activated charcoal can promote the rooting of tissue-cultured plantlets. Therefore, the optimal plant growth regulator concentration for the rooting of axillary buds in *Viburnum opulus* L. ‘Roseum’ was determined to be 1/2 WPM + 0.3 mg·L⁻^1^ NAA + 0.3 g·L⁻^1^ AC + 25 g·L⁻^1^ sucrose, with rooting beginning at approximately 15 days and the rooting rate reaching 88.89%.

The survival rate of transplanted tissue-cultured plantlets is a key factor in assessing whether the tissue culture technology system is mature. Light, temperature, substrate aeration, water retention, and nutrient capacity are important factors affecting the survival of tissue-cultured plantlets [24,25]. In this experiment, peat soil, vermiculite, and perlite were used as the substrates. The mixed substrate of peat soil, vermiculite, and perlite in a ratio of 1:2:1 was superior to other ratios for growth. The tissue-cultured plantlets of *Viburnum opulus* L. ‘Roseum’ exhibited deep green leaves and robust growth. This mixed substrate contained abundant moisture and nutrients, meeting the growth demands of *Viburnum opulus* L. ‘Roseum’ tissue-cultured plantlets.

## 4. Materials and Methods

### 4.1. Plant Materials

Using robust and disease-free *Viburnum opulus* L. ‘Roseum’ from the laboratory of Northeast Forestry University as the material, new spring branches from the current year were selected as explants to establish a tissue culture rapid propagation system.

### 4.2. Components of the Medium

The details of all the mentioned substances are as follows: Lloyd & McCown Woody Plant Basal Medium with Vitamins (WPM), L449; agar, PLANT TC, A111; 6-benzylaminopurine (6-BA) at a concentration of 1 mg·mL⁻^1^, T818; indole-3-butyric acid solution (IBA) at 1 mg·mL⁻^1^; naphthaleneacetic acid (NAA) at 1 mg·mL⁻^1^; activated carbon (AC), I460, from PhytoTechnology Laboratories; and sucrose, A502792 from Sangon Biotech (Shanghai, China). Peat soil was from Hongli Flower Base, Harbin, Heilongjiang Province, China.

### 4.3. Cultivation Conditions

The WPM medium base consisted of 5.3 g·L⁻^1^ plant agar, adjusted to a pH of 5.8 ± 0.02. Although the medium formulation remained consistent across various growth stages, the type and concentration of plant growth regulators differed. The medium was sterilized in an autoclave at 121 °C for 20 min. Incubation was maintained at a temperature of 25 ± 1 °C, with a light intensity of 40–50 μmol·m⁻^2^·s⁻^1^, following a 16/8 h light–dark cycle.

### 4.4. Sterilization of Explants

Healthy, pest-free, one-year-old fresh branches with bud points were cut and trimmed into small segments approximately 3.0 cm long, each containing a bud point. First, the material was washed in sterile water containing 2.0 mg·L^−1^ Tween 20 for 3–5 min and then covered with gauze and rinsed under running water for 30 min. After rinsing, the material was transferred to a vertical laminar flow cabinet for explant sterilization. The stem segments was washed once with sterile water and then immersed in 75% ethanol for 30 s while shaking thoroughly to ensure the alcohol made sufficient contact with the surface of the stem segments. After this, they were washed twice with sterile water and then disinfected with 2%, 3%, or 5% sodium hypochlorite for 5, 10, or 15 min. After disinfection, they were washed five times with sterile water. The disinfection treatment combinations are listed in Table 1. Finally, sterile filter paper was used to dry the moisture from the surface of the stem segments. Then, sterile scissors was used to trim off the ends that had been oxidized by the sodium hypochlorite. The remaining stem segments should be cut into small segments of about 1.5–2.0 cm and seeded into WPM minimal medium (each stem segment has two axillary buds). Each vial was inoculated with three stem segments and ten vials were replicated for each treatment group for a total of thirty tissue-cultured plantlets. The culture conditions were as follows: illumination for 16 h at an intensity of 40–50 μmol·m^−2^·s^−1^ and a temperature of 25 °C ± 1 °C. After 7 days, the rates of contamination, mortality, and survival of the stem segments were observed and counted as follows: contamination rate (%) = (total number of samples processed/number of contamination samples) × 100%; mortality rate (%) = (total number of treated samples/number of dead samples) × 100%; survival rate (%) = (total number of treated samples/number of surviving samples) × 100%.

### 4.5. Primary Culture of Viburnum opulus L. ‘Roseum’

The cleaned explants were inoculated onto WPM medium for axillary bud induction. Referring to Bi Xianyu’s [23] initial induction protocol, the following initial induction protocol was selected: WPM + 0.2 mg·L^−1^ IBA + 2.0 mg·L^−1^ 6-BA + 25 g·L^−1^ sucrose + 5.3 g·L^−1^ agar (A); WPM + 0.15 mg·L^−1^ IBA + 2.0 mg·L^−1^ 6-BA + 25 g·L^−1^ sucrose + 5.3 g·L^−1^ agar (B). Three explants were inoculated per bottle, with ten replicates for each protocol. The time of sprouting of axillary buds was counted, and the status of the sprouted axillary buds was observed within 45 days.

### 4.6. Induction of Bud Clusters from Young Stems

Using the induced sterile axillary buds as materials, they were transferred to media containing different combinations of plant growth regulators for proliferation culture. An L_9_ (3^4^) orthogonal experimental design was used, as shown in Table 5. Three sterile buds were inoculated per bottle, with ten replicates for each treatment. After 30 days of cultivation, the proliferation and growth status of the buds were observed and recorded.

### 4.7. Rooting Culture

The robust, sterile tissue-cultured shoots of *Viburnum opulus* L. ‘Roseum’ were transferred into a rooting culture medium using 1/2 WPM basic nutrient medium. Different concentrations of sucrose, NAA, and AC were designed using an L_9_ (3^4^) orthogonal experimental design for the rooting culture, as shown in Table 6. Each treatment was replicated in twelve bottles, with three plants per bottle. The rooting conditions were observed and recorded after 40 days.

### 4.8. Ex Vitro Acclimatization

Before transplanting, the caps of the tissue culture bottles were first loosened and then a small amount of sterile water was added. The plants were acclimatized indoors for 24 to 48 h. The plantlets were gently removed using tweezers, and the residual culture medium was rinsed from the root necks with tap water. They were transplanted into four substrates: a mixture of vermiculite and peat soil (1:1); a mixture of peat soil, vermiculite, and perlite (1:1:1); a mixture of peat soil and vermiculite (1:2); and a mixture of peat soil, vermiculite, and perlite (1:2:1). Three groups of ten tissue-cultured plantlets were planted in each substrate for a total of thirty plants. They were watered thoroughly once and covered with clear plastic cups to maintain humidity for 3 days. The cups were then removed. The survival rate was counted after 30 days.

### 4.9. Statistical Analysis

The experimental data are expressed as the mean ± standard error (SE) and were analyzed using an ANOVA followed by Duncan’s multiple range test in IBM SPSS Statistics 26.0. A significance level of *p* < 0.05 was established. Tables were created using Microsoft Excel 2010 and Microsoft Word 2020. The figures were produced in Adobe Photoshop 2020.

## 5. Conclusions

In this study, a complete rapid propagation technology for *Viburnum opulus* L. ‘Roseum’ was established, and the optimal culture conditions for different stages of tissue culture propagation were optimized. The best method for explant disinfection was found to be 75% ethanol for 30 s and 5% sodium hypochlorite for 5 min, resulting in a survival rate of 73.33%. The ideal medium for primary induction was WPM + 2.0 mg·L⁻^1^ 6-BA + 0.15 mg·L⁻^1^ IBA. The most effective proliferation scheme for adventitious buds was WPM + 1.0 mg·L⁻^1^ 6-BA + 0.15 mg·L⁻^1^ IBA + 25 g·L⁻^1^ sucrose, with a proliferation efficiency of 7.17. The most suitable rooting medium was 1/2 WPM + 0.3 mg·L⁻^1^ NAA + 0.3 mg·L⁻^1^ AC + 25 g·L⁻^1^ sucrose. The optimal transplant substrate was a 1:2:1 mixture of peat soil–vermiculite–perlite. The cultivation system effectively shortened the plantlet time and significantly improved the axillary bud induction rate and rooting rate of *Viburnum opulus* L. ‘Roseum’.

## Figures and Tables

**Table 1:** Effect of different treatment combinations on the sterilization of explants.

Group	Sodium Hypochlorite Concentration	Sodium Hypochlorite Treatment Time	Contamination Rate %	Mortality Rate %	Recovery Rate%
1	2%	5 min	80.00 ± 1.92 a	0.00	20.00 ± 3.33 d
2	2%	10 min	62.22 ± 1.11 b	0.00	37.78 ± 2.94 b
3	5%	5 min	24.44 ± 2.94 c	2.22 ± 1.92 d	73.33 ± 1.92 a
4	5%	10 min	21.11 ± 2.22 c	10.00 ± 1.92 c	67.78 ± 2.22 a
5	10%	5 min	13.33 ± 1.92 d	53.33 ± 6.67 b	28.89 ± 1.11 c
6	10%	10 min	0.00	75.56 ± 3.85 a	24.44 ± 1.11 cd

Note: Different lowercase letters in the same column indicate significant differences between treatments (*p* < 0.05).

**Table 2:** Time to sprouting of axillary buds and axillary bud status after 45 days for the two initial induction protocols.

Group	Number of Inoculated Stem Segments/pc	Axillary Bud Sprouting Time/Day	Status of Axillary Buds
A	30	8.67	Leaf blade curled, apical dominance not obvious, more deformed buds
B	30	10.33	Axillary buds grow well, with spreading, bright green leaves

**Table 3:** Effect of different concentrations of 6-BA, IBA, and sucrose on adventitious bud differentiation.

Groups	6-BA Concentration (mg L^−1^)	IBA Concentration (mg L^−1^)	Sucrose Concentration (g·L^−1^)	Multiplication Coefficient	Growth State of Tissue-Culture Plantlets
1	1.0	0.05	20	5.83 ± 0.31 abc	Leaves yellowing, differentiation buds turning red, growth is average.
2	1.0	0.1	30	3.67 ± 0.42 e	Few differentiation buds, signs of vitrification, stems and leaves are thick and red.
3	1.0	0.15	25	7.17 ± 0.48 a	More differentiation buds, leaf shape normal, growth is good.
4	1.5	0.05	30	4.17 ± 0.31 de	Growth is average, stems have hyperplastic tissue proliferation.
5	1.5	0.1	25	4.67 ± 0.49 cde	Growth is average, leaves are slender, leaf edges turning red.
6	1.5	0.15	20	3.83 ± 0.31 de	Leaves are slender, most differentiation buds are malformed.
7	2.0	0.05	25	5.17 ± 0.31 bcd	Growth is average, leaf edges slightly red, and some malformed buds present.
8	2.0	0.1	20	3.50 ± 1.0.43 e	Growth is average, fewer differentiation buds, leaves curling.
9	2.0	0.15	30	6.33 ± 0.766 ab	Growth is poor, leaves yellowing, more differentiation buds.

Note: Different lowercase letters in the same column indicate significant differences between treatments (*p* < 0.05).

**Table 4:** Effect of different concentrations of sucrose, NAA, and AC on rooting.

Groups	Sucrose Concentration (g·L^−1^)	NAA Concentration (mg L^−1^)	AC Concentration (mg L^−1^)	Rooting Rate %	Average Number of Primary Roots	Tissue-Culture Plantlets Grow and Root
1	25	0.1	0.1	23.46 ± 1.23 d	2.00 ± 50.58 d	Tissue-cultured plantlets grew poorly, browning at the base with only a few roots.
2	25	0.2	0.5	56.79 ± 3.27 c	2.80 ± 0.17 bc	Tissue-cultured plantlets have average growth with few roots.
3	25	0.3	0.3	88.89 ± 3.70 a	4.38 ± 0.08 a	Tissue-cultured plantlets have good growth with strong primary roots.
4	30	0.1	0.5	77.78 ± 4.28 b	4.29 ± 0.10 a	Tissue-cultured plantlets with good growth, weak primary root.
5	30	0.2	0.3	77.78 ± 3.70 b	2.29 ± 0.05 cd	Tissue culture plantlets were average in growth, with late root formation and short primary roots.
6	30	0.3	0.1	55.56 ± 4.28 c	3.40 ± 0.21 b	Tissue-culture plantlets have poor growth, with few main roots, thickened epidermis, and the growth of callus at the base.
7	35	0.1	0.3	55.56 ± 2.14 c	3.40 ± 0.10 b	Tissue-culture plantlets have average growth, with few roots and a small number of secondary roots.
8	35	0.2	0.1	56.79 ± 3.27 c	2.20 ± 0.07 cd	Tissue-culture plantlets have average growth, with a thickened epidermis on the main root.
9	35	0.3	0.5	55.56 ± 3.70 c	1.20 ± 0.05 e	Tissue-culture plantlets have poor growth, with weak and short main roots and browning at the base.

Note: Different lowercase letters in the same column indicate significant differences between treatments (*p* < 0.05).

**Table 5:** Table of orthogonal experimental design for axillary bud proliferation program.

Group	6-BA Concentration (mg·L^−1^)	IBA Concentration (mg·L^−1^)	Sucrose Concentration (g·L^−1^)
1	1.0	0.05	20
2	1.0	0.1	30
3	1.0	0.15	25
4	1.5	0.05	30
5	1.5	0.1	25
6	1.5	0.15	20
7	2.0	0.05	25
8	2.0	0.1	20
9	2.0	0.15	30

**Table 6:** Table of orthogonal experiments for rooting schemes.

Group	Sucrose Concentration (g·L^−1^)	NAA Concentration (mg·L^−1^)	AC Concentration (mg·L^−1^)
1	25	0.1	0.1
2	25	0.2	0.5
3	25	0.3	0.3
4	30	0.1	0.5
5	30	0.2	0.3
6	30	0.3	0.1
7	35	0.1	0.3
8	35	0.2	0.1
9	35	0.3	0.5

## Data Availability

All data are available in this manuscript.
